# Reducing Inter-Individual Differences in Task fMRI Preprocessing with OGRE (One-Step General Registration and Extraction) Preprocessing

**DOI:** 10.1007/s12021-025-09741-6

**Published:** 2025-09-30

**Authors:** Mark P. McAvoy, Lei Liu, Ruiwen Zhou, Benjamin A. Philip

**Affiliations:** 1 Program in Occupational Therapy, Washington University School of Medicine, St. Louis, MO USA; 2 Center for Biostatistics and Data Science, Washington University School of Medicine, St. Louis, MO USA

**Keywords:** Image analysis pipeline, fMRI, Brain, Software, Magnetic resonance imaging, Humans, Brain mapping

## Abstract

Volumetric analysis methods continue to enjoy great popularity in the analysis of task-related functional MRI (fMRI) data. Among these methods, omnipresent throughout the field is the software tool FEAT, part of the FSL package (FMRIB, Oxford, UK). However, FEAT preprocessing involves multi-step interpolation (multiple sequential transformations), which at typical fMRI field strengths (i.e. 3T) has not been systematically compared with with alternative methods, in part due to lack of software tools compatible with FSL FEAT analysis. Here we developed the One-step General Registration and Extraction (OGRE) pipeline to combine FreeSurfer brain extraction, FSL FNIRT registration, one-step interpolation of preprocessing transformations, and output for FSL FEAT analysis. We compared OGRE with two alternative preprocessing methods (fMRIPrep and FSL) in a dataset wherein adult human volunteers (*N* = 53) performed a precision drawing task during fMRI scanning. OGRE preprocessing led to lower inter-subject variability than FSL (*p* = 7.3 × 10^− 9^) or fMRIPrep (*p* = 0.036); OGRE also led to the strongest detection of task-related magnitude in primary motor cortex contralateral to movement (OGRE > FSL *p* = 4.2 × 10^− 4^, other method pairs not significant). This pattern suggests that one-step interpolation (used by OGRE and fMRIPrep) reduces inter-individual variability during moderate-resolution (2 mm^3^) volumetric analysis of task fMRI data.

**Supplementary Information:**

The online version contains supplementary material available at https://doi.org/10.1007/s12021-025-09741-6.

## Introduction

Numerous methods and software packages exist for the analysis of task fMRI data, and many of these approaches offer different advantages and tradeoffs. One commonly used analysis package is the fMRIB Software Library (FSL; Jenkinson et al., 2012), particularly its FEAT software for general linear model (GLM) volumetric analysis of fMRI data (Smith et al., 2004) which has over 15,000 citations, including over 2,400 since 2023 (Google Scholar, April 2025). However, FEAT preprocessing uses multi-step interpolation, in which each preprocessing step (e.g. motion correction) produces a transformed image that serves as input to the next step (e.g. registration). This chain of interpolations has the potential to induce unwanted spatial blurring, at least in high-field (7T) MRI (Polimeni et al., 2018; Renvall et al., 2016; Wang et al., 2022). However, the impact of multi-step interpolation on typical field strengths (i.e. 3T) remains unknown.

In contrast to multi-step interpolation, “one-step interpolation” uses a single procedure to correct for head motion, susceptibility distortion, intrasubject registration, and spatial normalization. One-step interpolation was developed for the Human Connectome Project (HCP) fMRI processing pipeline (Glasser et al., 2013), and is a small portion of their full processing pipeline, which subsequently uses a surface-based approach (Coalson et al., 2018; Dickie et al., 2019; Glasser et al., 2016, 2018). Another popular implementation of one-step interpolation for volumetric analysis is available via fMRIPrep (Esteban et al., 2019) with over 3000 citations (Google Scholar, April 2025). This package touts a modular design utilizing a variety of readily available software tools from sources including FSL, FreeSurfer (e.g. Fischl & Dale, 2000; Fischl et al., 2002; Fischl et al., 2004), AFNI (Cox, 1996), ANTs (Tustison et al., 2021), and others; however, fMRIPrep does not provide a complete alternative to FEAT because fMRIPrep is a preprocessing pipeline only, and thus must be integrated with additional tools for GLM analysis. Nevertheless, few studies have assessed the potential advantages of integrating alternative preprocessing piplelines into FSL FEAT for volumetric statistical analysis (Esteban et al., 2019). Direct tests that control for later processing steps have been limited because neither fMRIPrep nor the HCP pipeline are designed for off-the-shelf application to FSL FEAT (Mumford, 2017).

Here, we created the OGRE (One-step General Registration and Extraction) pipeline to integrate one-step interpolation and other volumetric-applicable components of HCP preprocessing (e.g. distortion and motion correction, registration and spatial normalization) as a general-purpose fMRI preprocessing tool for FSL FEAT statistical analysis. We performed a fully-controlled comparison of three preprocessing pipelines: OGRE, fMRIPrep and FSL FEAT preprocessing, by evaluating the three preprocessing pipelines on a fMRI motor task with subsequent volumetric FSL FEAT statistical analyses. The three preprocessing methods were compared for differences in inter-individual variability, task-related magnitude and brain extraction.

## Materials & Methods

### Participants

Fifty-three right-handed adults (38 female; ages 47 ± 18, range 22-82) performed a precision drawing task (see fMRI Task below) in the fMRI scanner. Participants were recruited for a different study; as a result, 18/53 participants had peripheral nerve injuries to their right arm, but the differences between groups (categorical or performance-related) were not a focus of the current study. Exclusion criteria included motor function diagnoses other than the aforementioned nerve injury (ongoing or in the prior 2 years), left handed or ambidextrous (self-report or Edinburgh Handedness Inventory < +40 (Oldfield, 1971)), major psychiatric/neurological diagnoses, chronic pain, and contraindications for MRI. Informed consent was obtained from all participants for being included in the study. All procedures were approved by the Institutional Review Board at Washington University in St. Louis School of Medicine.

### fMRI Acquisitions

Scans were performed on a Siemens (Erlangen, Germany) PRISMA 3T MRI scanner. BOLD EPIs for fMRI were collected using a T2*-weighted gradient echo sequence, a standard 64-channel birdcage radio-frequency coil, and the following parameters: TR = 662 ms, TE = 30 ms, flip angle = 52°, 72 × 72 voxel matrix, FOV = 216 mm, 60 contiguous axial slices acquired in interleaved order, resolution: 3.0 × 3.0 × 3.0 mm, bandwidth = 2670 Hz/pixel, multi-band acceleration = 6x. Siemens auto-align was run at the start of each session.

**Fig. 1:**
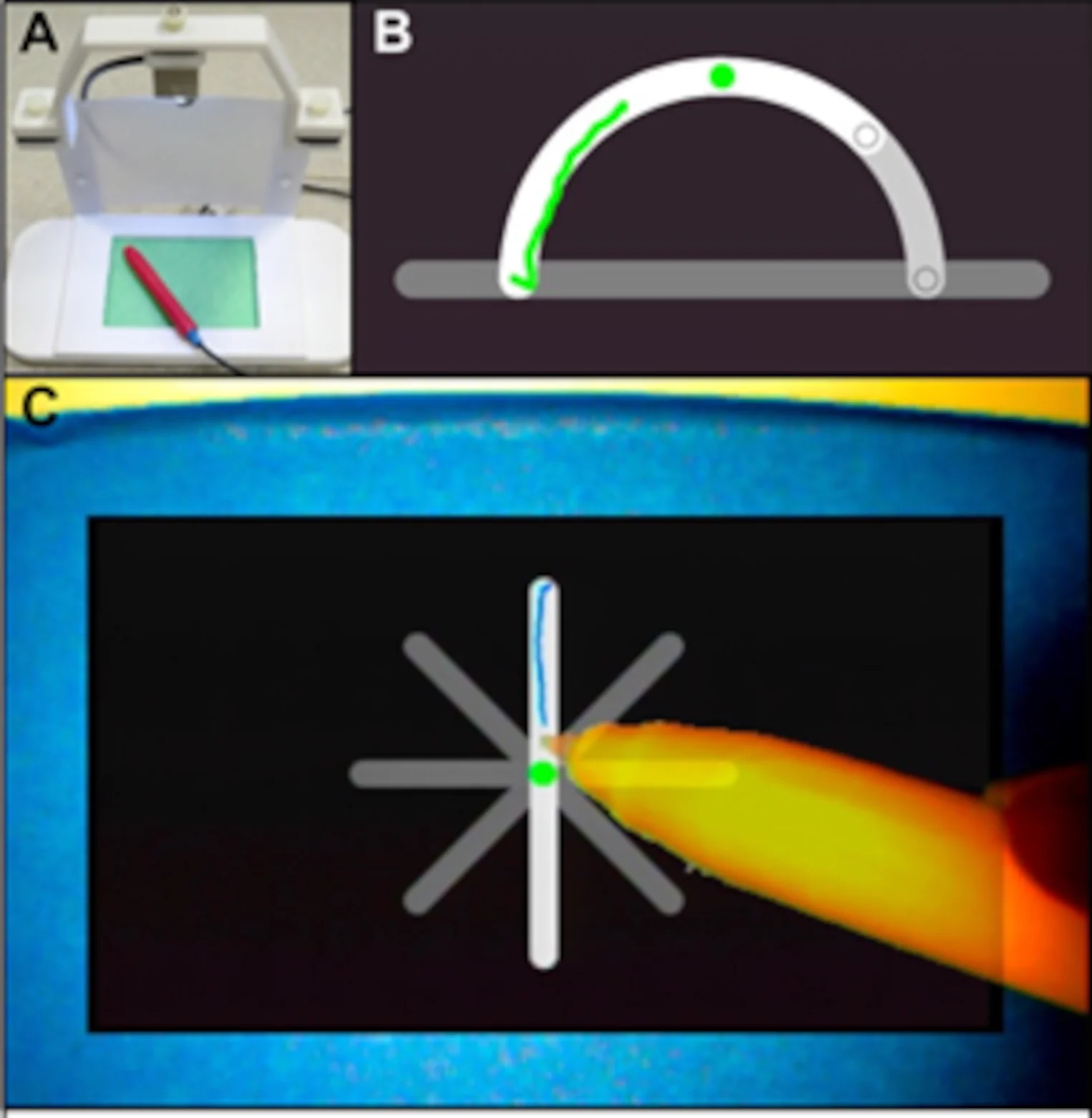
Motor task used for pipeline testing. **A**: MRI-compatible tablet. **B**: STEGA precision drawing task. **C**: Participant view of bluescreen integration

High-resolution T1-weighted structural images were also acquired, using the 3D MP-RAGE pulse sequence: TR = 4500 ms, TE = 3.16 ms, TI = 1000 ms, flip angle = 8.0°, 256 × 256 voxel matrix, FOV = 256 mm, 176 contiguous axial slices, resolution: 1.0 × 1.0 × 1.0 mm. A T2-weighted image was also acquired at: TR = 3000 ms, TE = 409 ms, 256 × 256 voxel matrix, FOV = 256 mm, 176 contiguous axial slices, resolution: 1.0 × 1.0 × 1.0 mm. Spin echo field maps were also collected before the functional runs.

### fMRI Task

Each participant completed 3 BOLD functional scans. During each scan, the participant used their right hand to perform a precision drawing task based on the STEGA app (Philip & Frey, 2014; Philip et al., 2023). (These runs were interleaved with 3 additional runs where the participants used their left hand to perform the same task, but these left hand runs are not included in the current study.) Participants saw hollow shapes, and were instructed to draw a line within the bounds of the shape, as fast as possible while prioritizing staying in-bounds over speed. Participants saw a video overlay of their (transparent) hand/pen over the drawn shape, using an MRI-compatible drawing tablet with bluescreen technology (Karimpoor et al., 2015), as shown in Fig. 1. The task was presented in a block design with 15.2 s (23 images) of drawing, followed by 15.2 s (23 images) of rest (fixation cross). A scan comprised ten cycles of draw/rest, with an additional rest block at the start, and 3.3 s (5 images) of additional rest after the final rest block, leading to a total duration of 5:23 (230 images of drawing, 258 rest) per scan.

### Data Preprocessing: One-step General Registration and Extraction (OGRE) Pipeline

OGRE (One-step General Registration and Extraction) was developed on Macintosh (Apple, Cupertino CA) and is based on version 3.27 of the HCP pipeline (https://github.com/Washington-University/HCPpipelines/tree/v3.27.0). Preprocessing steps include FreeSurfer parcellation and brain extraction, followed by motion correction, field map distortion correction, and warping to the 2 mm MNI atlas via a single transformation (i.e. “one-step interpolation”) using FSL FLIRT and FNIRT (Andersson et al., 2007) as detailed in Glasser et al. (2013). New features added for OGRE included the option to specify the native resolution as 0.7, 0.8, or 1.0 mm; the option to leave fMRI time series in native HCP space instead of registering to MNI atlas; extended compatibility to FreeSurfer versions 7.2.0, 7.3.2, 7.4.0 and 7.4.1 (https://surfer.nmr.mgh.harvard.edu/); adjustable dilation/erosion settings for brain masks; and the addition of three new steps to match FEAT: optional nonlinear spatial filtering (Smith & Brady, 1997), optional nonlinear temporal high pass filtering (Woolrich et al., 2001), and a switch from mean-based to median-based intensity normalization. OGRE also produces FEAT-formatted registration images and matrices for higher-level FEAT analysis. OGRE preprocessing outputs were entered as data for analysis in FEAT using its “Statistics” option (rather than “Full Analysis,” since OGRE has replaced FEAT preprocessing).

OGRE software and documentation are available online at: https://github.com/PhilipLab/OGRE-pipeline or https://www.nitrc.org/projects/ogre/.

#### Parallel Preprocessing Methods: OGRE vs. fMRIPrep vs. FSL-Preproc

**Fig. 2:**
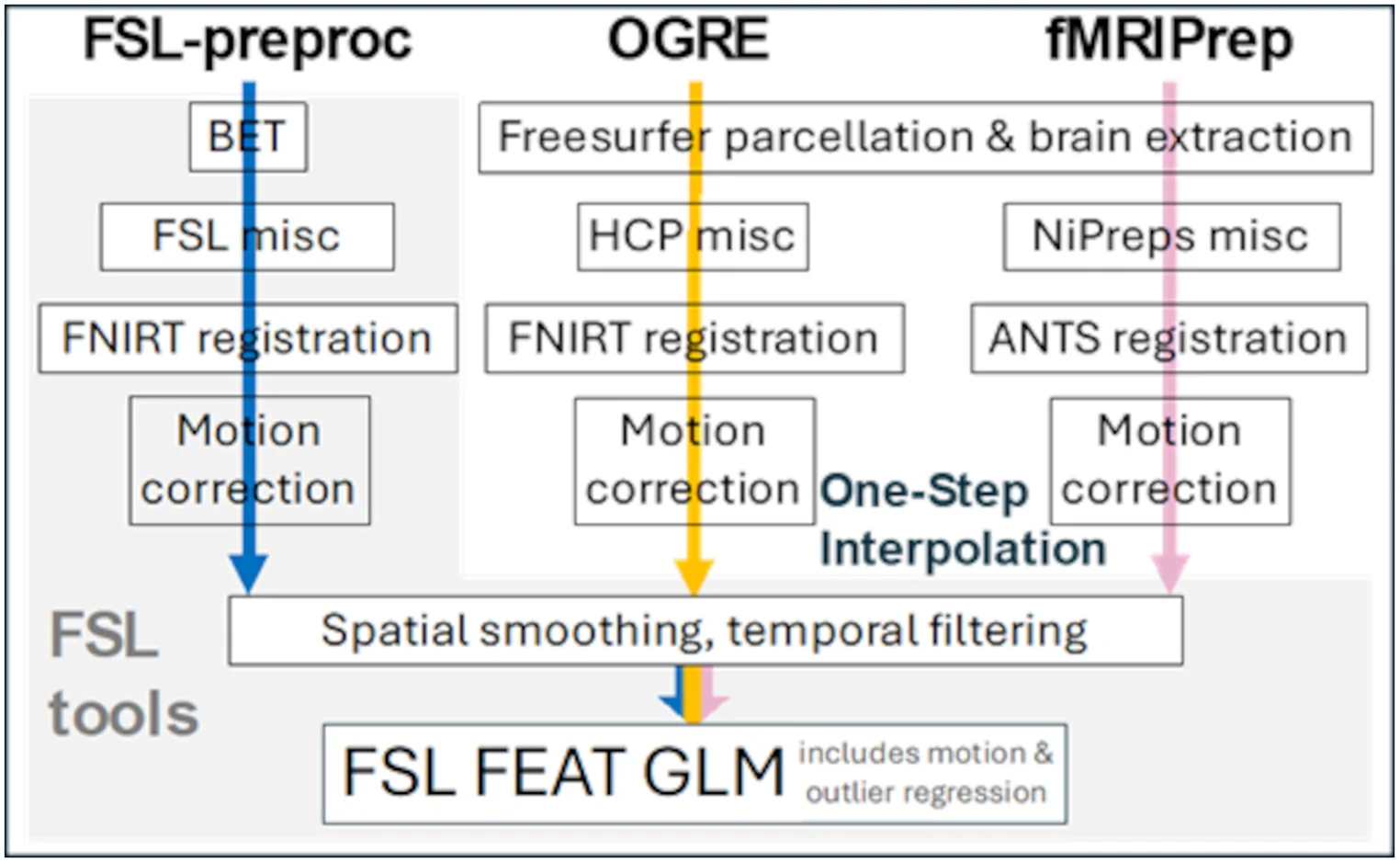
Overview of analysis methods. ‘Misc’ = EPI unwarp, intensity normalization, motion estimation. Registration = 2 mm MNI template. Spatial smoothing = 6 mm. Temporal filtering = high pass, 60 sec

We analyzed data using 3 parallel preprocessing methods, with the same subsequent FEAT GLM on the preprocessed data, as illustrated in Fig. 2. Note that all three methods are preprocessing, but we specify “FSL-preproc” to distinguish it from the full FSL pipeline.

In the “OGRE method,” the OGRE pipeline described above was used to preprocess data with the following options: FreeSurfer 7.4.1, native resolution of 1 mm, distortion correction using spin echo field maps, spatial smoothing kernel of 6 mm FWHM, intensity normalization via “grand median scaling” to a value of 10,000, high pass temporal filtering with 60 s cutoff, and output registration to 2 mm MNI atlas. Subsequently, GLM statistical analysis was performed with FEAT version 6.0.7 (Smith et al., 2004). Explanatory variables (EVs) were modeled, along with their temporal derivatives, according to the block design described in the previous section (*fMRI Task*), with additional confound EVs based on head motion parameters (translation and rotation). Volumes with excess head motion were addressed (Siegel et al., 2014) via additional confound EVs calculated within each time series as framewise displacement of 75th percentile + 1.5* interquartile range, using the FSL script FSLMotionOutliers. The hemodynamic response was accounted for by convolving the model with a double-gamma function. First-level contrasts of parameter estimates (COPEs) were calculated for Task vs. Rest. The first-level COPE (Task > Rest) served as input to higher-level analyses performed using a fixed-effects model. Z-statistic (Gaussianized T) images were thresholded using clusters determined by Z ≥ 3.1 and a corrected cluster significance threshold of *p* < 0.05. The first-level COPEs were averaged across runs for each participant (second level). Region of interest (ROI) analyses were performed on second-level (i.e. participant-level) data, as detailed in *ROI Analyses* below.

In the “fMRIPrep method,” preprocessing was performed using fMRIPrep 23.2.3 (Esteban et al., 2019), using default settings except that slice timing was ignored, sub-millimeter reconstruction option was disabled, and outputs were registered to the 2 mm MNI152NLin6Asym atlas (Grabner et al., 2006), the same atlas used by OGRE and FSL-preproc. Full fMRIPrep methods are described in Supplementary Methods. FSL was used to perform spatial smoothing with a 6 mm FWHM and high pass temporal filtering with 60 s cutoff, and registration outputs were modified following established workarounds for integrating fMRIPrep output with FSL (Mumford, 2017). Subsequently, GLM statistical analysis was performed with FEAT as described for the OGRE method above.

In the “FSL-preproc method,” preprocessing included the following steps: Non-brain structures were removed using BET. Head movement was reduced using MCFLIRT motion correction. Distortion correction was applied using spin echo field maps using FSL PRELUDE and FUGUE. Paralleling the OGRE implementation, spatial smoothing, intensity normalization, and the high-pass temporal filter were applied with the same settings as above. Functional data were registered with the high-resolution structural image using boundary-based registration (Greve & Fischl, 2009), and resampled to 2 × 2 × 2 mm resolution using FLIRT; the participant images were then registered to the 2 mm MNI152NLin6Asym atlas (Grabner et al., 2006) using FNIRT nonlinear registration (Andersson et al., 2007). Subsequently, GLM statistical analysis was performed with FEAT as described for the OGRE method above.

#### ROI Analyses

To quantitatively compare the three methods, a region-of-interest (ROI) approach was used, using an atlas of 300 volumetric ROIs with known network assignments (Seitzman et al., 2020). For each participant, the signal magnitude was calculated as the mean % BOLD signal change for the Draw > Rest contrast in second-level within-participant analyses.

The three methods were compared via two approaches. The first approach focused on inter-individual variability of signal magnitude, as measured by the standard deviation of the signal magnitudes across the 53 participants. Using a repeated measures analysis of variance (RMANOVA) with ROI as the random factor (i.e. equivalent to a “between-subjects” factor) and Method as a categorical within-subject factor, such that each method contributed 300 values, one per ROI. Significance was assessed at ⍺ = 0.05, and the Greenhouse-Geiser correction was applied to correct for non-sphericity of the data. Achieved power was calculated via a post hoc power analysis in G*Power 3.1.9.6, (Faul et al., 2007). When a trend or significant effect was observed, post-hoc comparisons were performed using Tukey’s Honestly Significant Difference (HSD). In addition, on a post-hoc basis, between-method correlations were calculated for each ROI as the Pearson *r* across the 53 subjects, and reported for each pair of methods as mean ± SD across ROI. The variability RMANOVA results were confirmed with a direct but unpaired test of variability via a Bartlett’s test of equal variances (MATLAB function “vartestn”).

The second approach for comparing methods focused on signal magnitude itself. This approach used a single task-related ROI (rather than the 300 ROIs) because in most brain areas, signal magnitude has no clear relationship with preprocessing quality (i.e. a better method could produce lower, higher, or unchanged magnitude). Instead, since participants used their right hand to perform the precision drawing task, a ROI was selected in the normatively defined left hemisphere hand primary motor cortex (M1) (Smith & Frey, 2011) as a sphere of 5 mm radius centered on MNI coordinates X = -38, Y = -24, Z = 54. This M1 ROI was used to compare the three methods by measuring each method’s Z-score for Draw > Rest at the top level (across participants). The Z-scores (i.e. each method had 53 values, one per participant) were analyzed following the same procedure as above, except that sphericity was not violated and thus not corrected.

On a post-hoc basis, additional RMANOVAs were used to analyze task-specific activity magnitude beyond M1. These RMANOVAs followed the methods in the previous paragraph, using ROIs in the left cortical hemisphere’s somatomotor network. 16 such ROIs existed in the Seitzman et al. (2020) atlas (ROI numbers 13, 15–27, 45–46), leading to 16 RMANOVAs and ⍺ = 3.1 × 10^− 3^ (0.05/16). Each ROI was labeled according to its centerpoint’s highest-probability area assignment in the Juelich Histological Atlas (Eickhoff et al., 2007); if the Juelich atlas assigned no area, a structural desciption was used from the Harvard-Oxford Cortical Structural Atlas (Desikan et al., 2006).

#### Whole-Brain Analyses

were carried out on the voxel-wise signal magnitudes. For each preprocessing method, a third-level analysis was implemented with an unpaired repeated measures FEAT GLM on the signal magnitudes (Draw > Rest) for each subject (i.e. second-level analysis). Subsequently, the three methods were compared via three pairwise repeated-measures FEAT GLMs, one for each pair of preprocessing methods, with significant clusters detected by Z ≥ 3.1 and a corrected cluster significance threshold of ⍺ = 0.0167 (0.05/3, Bonferroni correction for 3 GLMs).

#### Brain Extraction And Distortion Correction Comparisons

To quantify the difference in brain extraction and registration results between methods, we compared the size of post-registration (i.e. standard space) brain masks from each task fMRI scan, following Zhang et al. (2019). Specifically, for all three preprocessing methods, comparisons were performed on the reg_standard/mask.nii.gz files generated for the FEAT GLM. Voxels outside the brain were determined by subtracting the mask from the MNI152NLin6Asym standard template. Our two structural outcomes (number of voxels in mask, number of non-brain voxels in mask) were compared separately, using alpha = 0.025 (0.05/2 outcomes): first, effects of preprocessing method were identified with one-way ANOVAs; and second, comparisons with other outcome measures (BOLD standard deviation across ROIs, M1 z-score) were performed via a generalized linear mixed model (GLMM) on the random effect Participant and the within-participant factors Preprocessing Method, Mask Size, Non-Brain Voxels, and interactions involving preprocessing method.

Differences between methods in EPI distortion correction were not evaluated because all three methods perform distortion correction via FSL’s topup algorithm (Andersson et al., 2007; Esteban et al., 2019; Glasser et al., 2013; Smith et al., 2004). Fieldmap acquistions were available for all participants in this study.

**Fig. 3:**
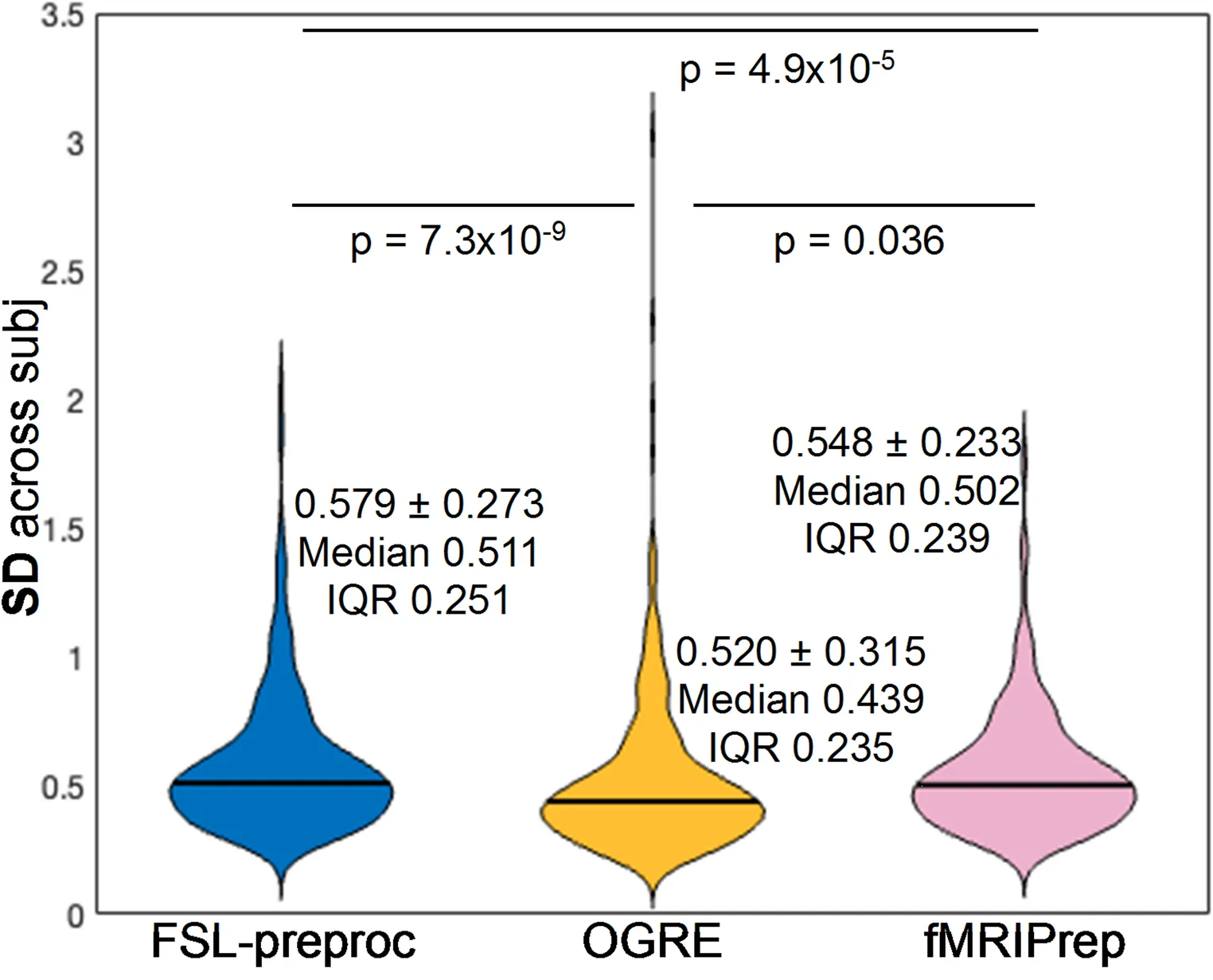
Lower inter-individual variability for OGRE than fMRIPrep or FSL-preproc. Each sample is a SD value across 53 participants; violin and statistics represent the distribution of samples across 300 ROIs. Main effect of Method (RMANOVA *p* = 1.8 × 10^-7^); *p*-values in figure are pairwise post-hoc tests

#### Participant Selection Effects

Because these participants were recruited for a different study, the participants included both typical adults and patients with upper extremity peripheral nerve injury. To determine whether this potential group effect introduced any biases between methods, we repeated our primary methods (RMANOVAs and post-hoc tests) on two alternative outcome variables for each ROI: between-groups difference in inter-individual variability (patient SD – typical SD) and between-groups difference in magnitude (patient mean – typical mean). In both, Greenhouse-geiser correction was applied to correct for non-sphericity.

## Results

### OGRE Preprocessing Led to Lower Inter-Individual Variability than FSL-Preproc or fMRIPrep

OGRE produced the lowest inter-individual variability as measured by the standard deviation across ROIs, followed by fMRIPrep, as shown in Fig. 3. An RMANOVA on the within-subject factor Method found a significant effect (F(2,598) = 18.87, *p* = 1.755 × 10^− 7^), reflecting a medium effect size ($\:{\eta\:}_{partial}^{2}$ = 0.059). This effect was driven by significant differences between all pairs of methods despite moderate to high correlations across methods (FSL-preproc vs. OGRE *r* = 0.80 ± 0.13, FSL-preproc vs. fMRIPrep *r* = 0.85 ± 0.10, OGRE vs. fMRIPrep *r* = 0.79 ± 0.12). These results were confirmed by a Bartlett’s test of equal variances, which found a significant effect of Method (*p* = 1.52 × 10-6) with significant differences between all pairs of methods (*p* ≤ 0.014). For a full list of results for each of the 300 ROIs, see Supplementary Table 1.

### OGRE, FSL-Preproc, and fMRIPrep Show Similar Sensitivity to Task-Related Activity Across Methods

**Fig. 4:**
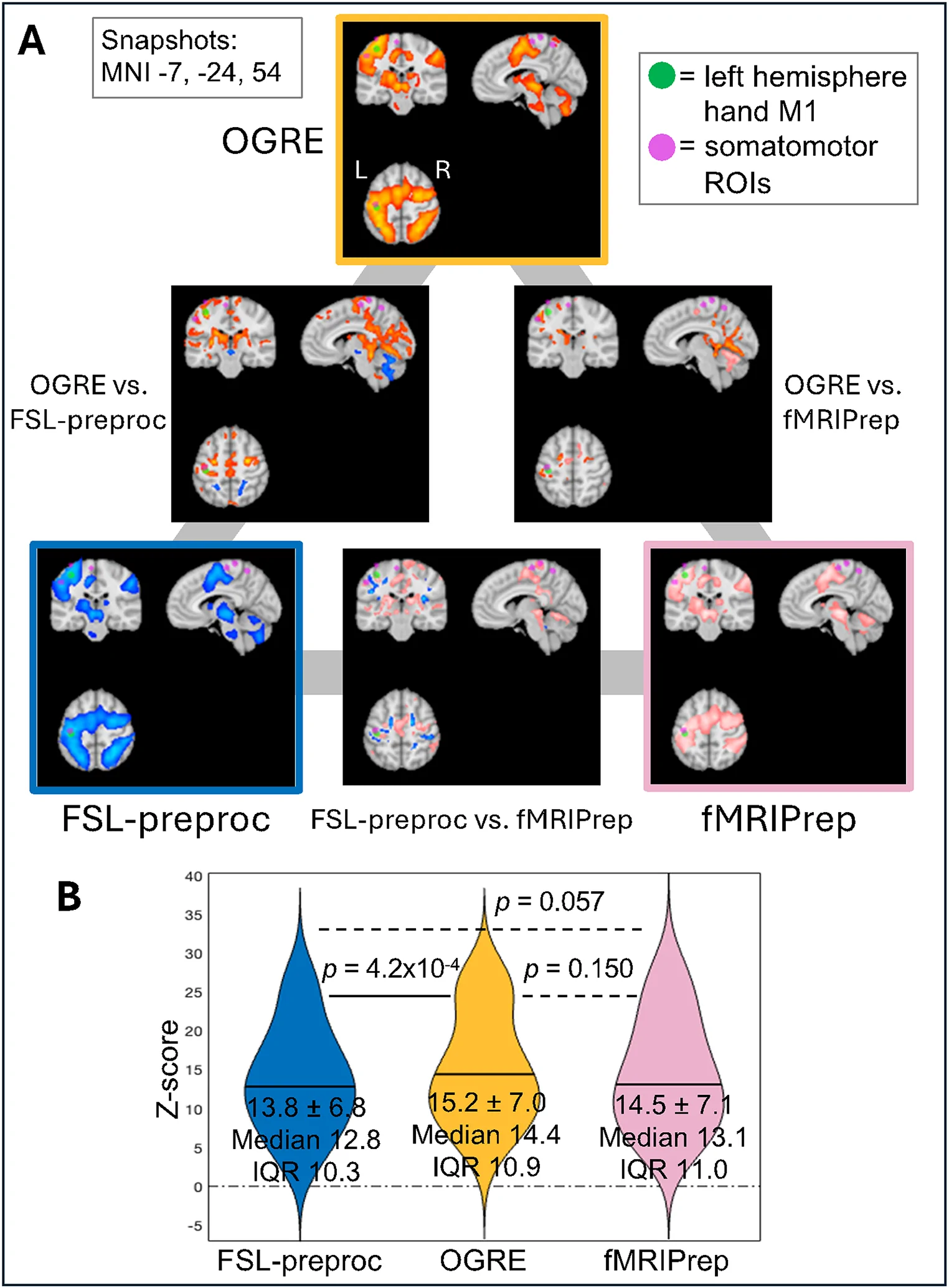
All methods lead to similer BOLD signal magnitudes for right hand drawing (Draw >Rest). **A**: Whole-brain third level (across participants) results for each analysis contrast (Z ≥ 3.1, cluster corrected). **B**: Z-scores in contralateral hand M1 (green circle in A) differ significantly between FSL-preproc and OGRE. Main effect of method (RMANOVA* p* = 3.1 × 10^-4^); *p*-values in figure are pairwise post-hoc tests

To assess the effects of the preprocessing methods over the whole brain, we conducted voxel-wise repeated measures analyses, treating the three methods (OGRE, fMRIPrep, FSL-preproc) as separate measures for each participant. Each method led to grossly similar results (Fig. 4A triangular array endpoints), but between-method contrasts showed significant differences between methods (Fig. 4A triangular array sides).

To provide a quantitative contrast between preprocessing methods in an area with known “ground truth,” we compared left hemisphere normative hand M1, contralateral to the drawing right hand (Fig. 4B). The Z-scores for Draw > Rest across subjects were highly correlated between methods (0.89 ≤ *r* ≤ 0.91 for any pair of methods), and a one-way RMANOVA found a significant effect of Method (F(2,72) = 8.75, *p* = 3.1 × 10^− 4^). This represented a large effect size ($\:{\eta\:}_{partial}^{2}$ = 0.144). Post-hoc tests indicated that this trend arose from lower Z-scores for FSL-preproc than OGRE (*p* = 4.2 × 10^− 4^), and a trend toward lower Z-scores for FSL-preproc than fMRIPrep (*p* = 0.057). OGRE Z-scores were numerically higher than fMRIPrep but this was not statistically significant (*p* = 0.150).

To ensure that these results were not M1-specific, we performed additional RMANOVAs using the Z-score from each the 16 left hemisphere somatomotor ROIs in the Seitzman et al. (2020) atlas. We found significant effects of Method in 7 of the ROIs (Table 1), and post-hoc tests revealed a marginal advantage for OGRE over fMRIPrep (OGRE > fMRIPrep in two ROIs, fMRIPrep > OGRE in one ROI), as shown in Table 1. Of the 7 ROIs with significant effects, 5 were in M1; of those five, the highest Z-scores were produced by OGRE in 3/5 ROIs, fMRIPrep in 1/5, and FSL in 1/5.

Overall, these results demonstrate that OGRE provides the most sensitive detection of task-related magnitude, with clear advantages over FSL-preproc and marginal advantages over fMRIPrep.

**Table 1:** Left hemisphere somatomotor ROIs (Seitzman et al., 2020). Method significantly affected z-score in 7/16 rois. α = 3.1 × 10^− 3^. M1 = primary motor; S1 = primary somatosensory

ROI center (MNI x, y, z)	Brodmann cortical area	RMANOVA stats		Significant post-hoc comparisons
		F	*p*	
-7, -52, 61	7A (parietal), 3b (S1)	0.64	0.506	–
-54, -23, 43	2 (S1)	10.25	2.58 × 10^− 4^	FSL-preproc > OGRE
-45, -32, 47	Inferior temporal	6.50	5.30 x -10^− 3^	–
-40, -19, 54	4 (M1)	20.94	1.11 × 10^− 7^	fMRIPrep > FSL-preproc fMRIPrep > OGRE
-38, -27, 69	4 (M1)	6.13	3.06 × 10^− 3^	FSL-preproc > OGRE
-38, -15, 69	Precentral gyrus	4.76	0.010	–
-29, -43, 61	1, 2 (S1)	1.75	0.185	–
-23, -30, 72	6 (premotor)	4.76	0.010	–
-21, -31, 61	4 (M1)	11.15	1.19 × 10^− 4^	OGRE > FSL-preproc OGRE > fMRIPrep fMRIPrep > FSL-preproc
-16, -46, 73	5 (parietal)	0.84	0.436	–
-14, -18, 40	Precentral gyrus	2.65	0.076	–
-13, -17, 75	6 (premotor)	1.49	0.231	–
-7, -21, 65	6 (premotor)	6.72	1.80 × 10^− 3^	OGRE > FSL-preproc
-7, -33, 72	4 (M1)	13.53	1.52 × 10^− 5^	OGRE > FSL-preproc fMRIPrep > FSL-preproc
-53, -10, 25	3b (S1)	3.22	0.057	–
-49, -11, 35	4 (M1)	9.45	1.69 × 10^− 4^	OGRE > fMRIPrep

### OGRE Identified Smaller Brain Volumes, but Brain Volume Size did not Affect Outcomes

To quantify the difference in brain extraction results between preprocessing methods, we compared the number of voxels in the post-registration (i.e. standard space) brain masks of the drawing runs (Table 2). Method had a significant effect on the number of voxels (ANOVA F_2,156 _= 299.85, *p* = 3.57 × 10^− 54^). Post-hoc analyses showed significant differences between all pairs, with mask size OGRE < fMRIPrep < FSL-preproc. We found the same pattern of results for number of voxels outside the brain (F_2,156 _= 337.35, *p* = 2.22 × 10^− 57^). However, analysis outcomes did not depend on mask size: we used GLMMs to evaluate the effects of Model, # Mask Voxels, and # Non-Brain Voxels on our two outcome measures (variability via BOLD standard deviation across ROIs, and magnitude via M1 Z-score), and we found no significant effects (*p* ≥ 0.35, for details see Supplementary Table 2).

**Table 2:** Sizes of brain masks after registration to MNI-152 standard space

	Total Voxels			Non-Brain Voxels		
Method	Mean ± SD	Median	Range	Mean ± std	Median	Range
FSL-preproc	311,110 ± 9,856	309,877	291,471 − 334,468	83,154 ± 9,705	81,789	63,314 − 106,250
OGRE	268,335 ± 6,303	267,491	260,819 − 290,348	41,318 ± 5,903	40,247	34,333 − 62,449
fMRIPrep	274,173 ± 12,184	272,907	227,179 – 305,691	48,020 ± 10,438	46,615	29,310 − 78,063

### Participant Selection does not Explain OGRE’s Advantages

Our participant sample included at least one major source of variance not included in our models: the distinction between typical adults and patients with peripheral nerve injury. This provides an advantage for practical testing, because it requires robustness in the face of complex real-world data with potentially unidentified sources of variance. However, we must evaluate whether this sample choice biased our results. To address this, we used a RMANOVA to assess whether preprocessing method affected between-groups differences in inter-individual variability or between-groups differences in magnitude of task-related BOLD signal in each ROI. For between-groups differnences in inter-individual variability, we found a significant effect of method (F_2,598 _= 4.88, *p* = 0.016), which was driven only by a significantly higher between-groups difference for OGRE than fMRIPrep (*p* = 0.018), but other pairs of methods did not differ (*p* > 0.15). In other words, group had a larger influence on inter-individual variability for OGRE than fMRIPrep. For group differences in signal magnitude, we found no effect of method (F_2,598 _= 1.32, *p* = 0.267). Out of an abdundance of caution, we also repeated our main analyses with only typical adult participants (*n* = 34; Supplementary Results) and found the same pattern of results as in our full dataset. Therefore, our unanalyzed effect of participant group did not systematically influence our comparisons between OGRE and FSL-preproc; and if participant group influenced our comparisons between OGRE and fMRIPrep, it did so by undercutting rather than inflating OGRE’s apparent advantages.

## Discussion

We compared a new fMRI preprocessing method, OGRE, with the established methods FSL and fMRIPrep. Following preprocessing, all three methods underwent identical statistical analyses via FSL FEAT to compare inter-individual variability and task-related magnitude during a precision drawing task. OGRE preprocessing led to lower inter-individual variability than fMRIPrep and FSL, and increased detection of task-related magnitude (Draw > Rest in contralateral hand M1) compared to FSL. The advantages of fMRIPrep and OGRE over FSL-preproc demonstrate that the two methods’ common features (FreeSurfer and one-step interpolation) may provide substantial benefits for volumetric preprocessing of task fMRI data. The small additional advantages of OGRE over fMRIPrep results suggest that OGRE’s unique combination of methods (one-step interpolation + FNIRT registration) may add further benefits for datasets similar to the one used here.

### Reduced Inter-Individual Variability after One-Step Interpolation and FNIRT Registration

Preprocessing method had a significant effect on inter-individual variability, with differences between all pairs of methods: lowest variability for OGRE, followed closely by fMRIPrep, each of which led to lower variability than FSL-preproc (Fig. 3). This suggests that one-step interpolation (simultaneous distortion correction, motion correction, registration, and spatial normalization) provided a significant advantage over FSL’s multi-step interpolation. Inter-individual variability is a longstanding challenge in fMRI research (Dubois & Adolphs, 2016; Van Horn et al., 2008) because group averaging creates a tradeoff between statistical power and relevance to individual participants. Numerous methods exist to tackle inter-individual variability in fMRI data, but the best methods require specific data collection tools or lengthy data collection sessions (Gordon et al., 2017; Michon et al., 2022; Napadow et al., 2008), so analysis-based approaches will remain useful in many situations.

Our finding of lower variability for OGRE than fMRIPrep suggests that other differences in preprocessing methods can also impact fMRI study outcomes, in combination with one-step interpolation. OGRE and fMRIPrep used the same tools for head motion estimation and correction (FSL MCFLIRT) and distortion correction (FSL topup), but different tools for spatial normalization (OGRE: FSL FNIRT, fMRIPrep: ANTs antsRegistration) (Esteban et al., 2019). Previous studies have demonstrated the two spatial normalization tools offer different tradeoffs: fMRIPrep’s ANTs has higher precision in capturing anatomical variations, whereas OGRE’s FNIRT better accommodates local deformations and atypicalities (Svejda et al., 2024). Our results suggest that (when combined with one-step interpolation) FNIRT’s accommodation of local deformations improves inter-subject comparisons, even in a dataset of brains without neurological disorders; and that ANTs’ high-precision advantage may provide limited value for moderate-resolution analyses such as the 2 mm^3^ resolution used here (collected at 3 mm^3^) and frequently in task-based volumetric studies.

### One-Step Interpolation Marginally Increases Detection of Task-Related Signal in Functional Analysis

Preprocessing method had a significant effect on detection of task-related magnitude as quantified by the Z-score in M1 contralateral to the drawing hand, but the effects were more subtle than for inter-individual variability: post-hoc tests revealed a pairwise difference only between OGRE (highest M1 Z-score) and FSL (lowest M1 Z-score). This effect was not an artifact of hand M1; we found a similar pattern of marginal adantages for OGRE > fMRIPrep > FSL in other areas of the somatomotor network contralateral to movement. These results suggest that both OGRE-and-fMRIPrep common features (e.g. one-step interpolation) and OGRE-specific features (e.g. the addition of FNIRT registration) contribute to the detection of BOLD task-related magnitude, but not all decreases in inter-subject variability lead to greater detection of task-related magnitude.

### The Precision of Brain Extraction is not a Critical Driver of Analysis Outcomes

FSL FEAT’s preprocessing has been criticized for its reliance on the Brain Extraction Tool (BET; Smith, 2002) to identify brain voxels in the scan volume, despite BET’s pervasiveness with over 12,000 citations (Google Scholar, April 2025). BET is prone to under- and over-extraction of brain voxels (Boesen et al., 2004; Klein et al., 2010; Novosad et al., 2018; Zhuang et al., 2006), and has limited options for adjusting its algorithmic criteria (Jenkinson, 2018). As a result, BET either produces suboptimal brain extractions or requires time-consuming and error-prone manual adjustment (Mohapatra et al., 2023; Quilis-Sancho et al., 2020). Although OGRE’s FreeSurfer-based brain extraction led to smaller brain volume estimates than fMRIPrep’s ANTs (Avants et al., 2011), which in turn were smaller than those of FSL-preproc’s BET, the smaller volumes did not affect inter-subject variability or detection of task-related magnitude. The lack of a brain-mask-size effect suggests that improved brain extraction does not necessarily play a critical role in the outcome of task fMRI analysis. Brain extraction accuracy may play a minor role, or a role in other metrics (Antonopoulos et al., 2023; Novosad et al., 2018); but here, the inclusion of non-brain voxels did not impair analysis outcomes, speculatively because of the lack of task-related BOLD signal in distal non-brain voxels.

### Use Case of OGRE: Improved Preprocessing for Researchers Who Prefer Volumetric Analysis

OGRE uses code and concepts developed by the Human Connectome Project (HCP), including the one-step interpolation (Glasser et al., 2013); but OGRE should not be mistaken for an implementation of the HCP pipeline, which incorporates many other features including a surface-based approach to fMRI data analysis (Dickie et al., 2019; Glasser et al., 2016, 2018). Surface-based analysis has many demonstrated benefits (Coalson et al., 2018), but not all analysis approaches are suitable for all research questions, groups, and protocols. Here we have quantified the advantages of FreeSurfer, FNIRT registration, and one-step interpolation; and demonstrated that those advantageous preprocessing steps can be leveraged to improve task fMRI analyses with the popular, accessible FSL and FreeSurfer toolboxes.

### Limitations

Here we compared three preprocessing methods using a precision drawing motor task fMRI dataset and two traditional volumetric magnitude-based analysis methods (ROI and whole-brain). Many future directions present themselves, including using multivariate similarity-based fMRI analyses (Nili et al., 2014); and evaluating other data sets such as other tasks, task-free fMRI and large datasets. Given the reduced inter-individual variability provided by OGRE preprocessing, it may demonstrate benefits across a broad range of analyses and datasets, and we welcome further testing of OGRE’s robustness.

OGRE version 1.X uses code derived from HCP 3.27.0, and as such may not reflect updates or bug fixes in future versions of the HCP code. Future updates to OGRE will link it to the main HCP repository to avoid this problem.

## Conclusion

Volumatric analysis of task fMRI data can be improved with preprocessing approaches that integrate tools from FSL, HCP, and FreeSurfer. We developed the OGRE (One-step General Registration and Extraction) software to provide FSL users with an off-the-shelf general-purpose implementation of FNIRT registration and one-step interpolation. We demonstrated that OGRE reduces inter-individual variability and increases signal magnitude in task-related regions of the brain, compared to preprocessing with FSL or fMRIPrep. The additional advantage OGRE over fMRIPrep (both of which use one-step interpolation) may reflect the tradeoffs between registration via FNIRT (FSL and OGRE) vs. ANTs (fMRIPrep) with medium-resolution fMRI data (3 mm^3^ collection, 2 mm^3^ analysis). OGRE consistently produced the best outcomes for volumetric analysis of our motor task fMRI data. OGRE is available online at https://github.com/PhilipLab/OGRE-pipeline or https://www.nitrc.org/projects/ogre/.

## Supplementary Information

Below is the link to the electronic supplementary material.

Supplementary Material 1

## Data Availability

The data that support the findings of this study are available on request from the corresponding author, BAP, upon reasonable request. The data will be made openly available upon completion of clinical trial NCT05207878.
