# Supplementary material for: Reducing Inter-Individual Differences in Task fMRI Preprocessing with OGRE (One-Step General Registration and Extraction) Preprocessing
Source: Neuroinformatics. 2025 Sep 30;23(4):47. doi: 10.1007/s12021-025-09741-6 (PMC12484288; doi:10.1007/s12021-025-09741-6)
Supplement: Supplementary file 1 — Supplementary Material 1 [file 12021_2025_9741_MOESM1_ESM.docx]

**Supplementary Methods**

The following text describes a single subject, but is identical for all subjects.

fMRIprep results included in this manuscript come from preprocessing performed using *fMRIPrep* 24.1.1 (Esteban et al. (2019); Esteban et al. (2018); RRID:SCR_016216), which is based on *Nipype*1.8.6 (K. Gorgolewski et al. (2011); K. J. Gorgolewski et al. (2018); RRID:SCR_002502).

**Preprocessing of B_0_ inhomogeneity mappings**

A total of 2 fieldmaps were found available within the input BIDS structure for this subject. A *B_0_*-nonuniformity map (or *fieldmap*) was estimated based on two (or more) echo-planar imaging (EPI) references with topup (Andersson, Skare, and Ashburner (2003); FSL None).

**Anatomical data preprocessing**

A total of 1 T1-weighted (T1w) images were found within the input BIDS dataset. The T1w image was corrected for intensity non-uniformity (INU) with N4BiasFieldCorrection (Tustison et al. 2010), distributed with ANTs 2.5.3 (Avants et al. 2008, RRID:SCR_004757), and used as T1w-reference throughout the workflow. The T1w-reference was then skull-stripped with a*Nipype* implementation of the antsBrainExtraction.sh workflow (from ANTs), using OASIS30ANTs as target template. Brain tissue segmentation of cerebrospinal fluid (CSF), white-matter (WM) and gray-matter (GM) was performed on the brain-extracted T1w using fast (FSL (version unknown), RRID:SCR_002823, Zhang, Brady, and Smith 2001). Brain surfaces were reconstructed using recon-all (FreeSurfer 7.3.2, RRID:SCR_001847, Dale, Fischl, and Sereno 1999), and the brain mask estimated previously was refined with a custom variation of the method to reconcile ANTs-derived and FreeSurfer-derived segmentations of the cortical gray-matter of Mindboggle (RRID:SCR_002438, Klein et al. 2017). A T2-weighted image was used to improve pial surface refinement. Brain surfaces were reconstructed using recon-all (FreeSurfer 7.3.2, RRID:SCR_001847, Dale, Fischl, and Sereno 1999), and the brain mask estimated previously was refined with a custom variation of the method to reconcile ANTs-derived and FreeSurfer-derived segmentations of the cortical gray-matter of Mindboggle (RRID:SCR_002438, Klein et al. 2017). Volume-based spatial normalization to three standard spaces (MNI152NLin6Asym, MNI152NLin2009cSym, MNI152NLin2009cAsym) was performed through nonlinear registration with antsRegistration (ANTs 2.5.3), using brain-extracted versions of both T1w reference and the T1w template. The following template was selected for spatial normalization and accessed with *TemplateFlow* (24.2.0, Ciric et al. 2022): *FSL’s MNI ICBM 152 non-linear 6th Generation Asymmetric Average Brain Stereotaxic Registration Model* [Evans et al. (2012), RRID:SCR_002823; TemplateFlow ID: MNI152NLin6Asym].

**Functional data preprocessing**

For each of the 6 BOLD runs found per subject (across all tasks and sessions), the following preprocessing was performed. First, a reference volume was generated, using a custom methodology of *fMRIPrep*, for use in head motion correction. Head-motion parameters with respect to the BOLD reference (transformation matrices, and six corresponding rotation and translation parameters) are estimated before any spatiotemporal filtering using mcflirt (FSL , Jenkinson et al. 2002). The estimated *fieldmap* was then aligned with rigid-registration to the target EPI (echo-planar imaging) reference run. The field coefficients were mapped on to the reference EPI using the transform. The BOLD reference was then co-registered to the T1w reference using bbregister (FreeSurfer) which implements boundary-based registration (Greve and Fischl 2009). Co-registration was configured with six degrees of freedom. The aligned T2w image was used for initial co-registration.Several confounding time-series were calculated based on the *preprocessed BOLD*: framewise displacement (FD), DVARS and three region-wise global signals. FD was computed using two formulations following Power (absolute sum of relative motions, Power et al. (2014)) and Jenkinson (relative root mean square displacement between affines, Jenkinson et al. (2002)). FD and DVARS are calculated for each functional run, both using their implementations in *Nipype* (following the definitions by Power et al. 2014). The three global signals are extracted within the CSF, the WM, and the whole-brain masks. Additionally, a set of physiological regressors were extracted to allow for component-based noise correction (*CompCor*, Behzadi et al. 2007). Principal components are estimated after high-pass filtering the *preprocessed BOLD* time-series (using a discrete cosine filter with 128s cut-off) for the two *CompCor* variants: temporal (tCompCor) and anatomical (aCompCor). tCompCor components are then calculated from the top 2% variable voxels within the brain mask. For aCompCor, three probabilistic masks (CSF, WM and combined CSF+WM) are generated in anatomical space. The implementation differs from that of Behzadi et al. in that instead of eroding the masks by 2 pixels on BOLD space, a mask of pixels that likely contain a volume fraction of GM is subtracted from the aCompCor masks. This mask is obtained by dilating a GM mask extracted from the FreeSurfer’s *aseg* segmentation, and it ensures components are not extracted from voxels containing a minimal fraction of GM. Finally, these masks are resampled into BOLD space and binarized by thresholding at 0.99 (as in the original implementation). Components are also calculated separately within the WM and CSF masks. For each CompCor decomposition, the *k* components with the largest singular values are retained, such that the retained components’ time series are sufficient to explain 50 percent of variance across the nuisance mask (CSF, WM, combined, or temporal). The remaining components are dropped from consideration. The head-motion estimates calculated in the correction step were also placed within the corresponding confounds file. The confound time series derived from head motion estimates and global signals were expanded with the inclusion of temporal derivatives and quadratic terms for each (Satterthwaite et al. 2013). Frames that exceeded a threshold of 0.388734 mm FD or 1.5 standardized DVARS were annotated as motion outliers. Additional nuisance timeseries are calculated by means of principal components analysis of the signal found within a thin band (*crown*) of voxels around the edge of the brain, as proposed by (Patriat, Reynolds, and Birn 2017). All resamplings can be performed with *a single interpolation step* by composing all the pertinent transformations (i.e. head-motion transform matrices, susceptibility distortion correction when available, and co-registrations to anatomical and output spaces). Gridded (volumetric) resamplings were performed using nitransforms, configured with cubic B-spline interpolation.

Many internal operations of *fMRIPrep* use *Nilearn* 0.10.4 (Abraham et al. 2014, RRID:SCR_001362), mostly within the functional processing workflow. For more details of the pipeline, see [the section corresponding to workflows in *fMRIPrep*’s documentation](https://fmriprep.readthedocs.io/en/latest/workflows.html).

**References (Supplementary Methods)**

Abraham, Alexandre, Fabian Pedregosa, Michael Eickenberg, Philippe Gervais, Andreas Mueller, Jean Kossaifi, Alexandre Gramfort, Bertrand Thirion, and Gael Varoquaux. 2014. “Machine Learning for Neuroimaging with Scikit-Learn.” *Frontiers in Neuroinformatics* 8. <https://doi.org/10.3389/fninf.2014.00014>.

Andersson, Jesper L. R., Stefan Skare, and John Ashburner. 2003. “How to Correct Susceptibility Distortions in Spin-Echo Echo-Planar Images: Application to Diffusion Tensor Imaging.”*NeuroImage* 20 (2): 870–88. <https://doi.org/10.1016/S1053-8119(03)00336-7>.

Avants, B. B., C. L. Epstein, M. Grossman, and J. C. Gee. 2008. “Symmetric Diffeomorphic Image Registration with Cross-Correlation: Evaluating Automated Labeling of Elderly and Neurodegenerative Brain.” *Medical Image Analysis* 12 (1): 26–41. <https://doi.org/10.1016/j.media.2007.06.004>.

Behzadi, Yashar, Khaled Restom, Joy Liau, and Thomas T. Liu. 2007. “A Component Based Noise Correction Method (CompCor) for BOLD and Perfusion Based fMRI.” *NeuroImage* 37 (1): 90–101. <https://doi.org/10.1016/j.neuroimage.2007.04.042>.

Ciric, R., William H. Thompson, R. Lorenz, M. Goncalves, E. MacNicol, C. J. Markiewicz, Y. O. Halchenko, et al. 2022. “TemplateFlow: FAIR-Sharing of Multi-Scale, Multi-Species Brain Models.” *Nature Methods* 19: 1568–71. <https://doi.org/10.1038/s41592-022-01681-2>.

Dale, Anders M., Bruce Fischl, and Martin I. Sereno. 1999. “Cortical Surface-Based Analysis: I. Segmentation and Surface Reconstruction.” *NeuroImage* 9 (2): 179–94. <https://doi.org/10.1006/nimg.1998.0395>.

Esteban, Oscar, Ross Blair, Christopher J. Markiewicz, Shoshana L. Berleant, Craig Moodie, Feilong Ma, Ayse Ilkay Isik, et al. 2018. “fMRIPrep 24.1.1.” *Software*. <https://doi.org/10.5281/zenodo.852659>.

Esteban, Oscar, Christopher Markiewicz, Ross W Blair, Craig Moodie, Ayse Ilkay Isik, Asier Erramuzpe Aliaga, James Kent, et al. 2019. “fMRIPrep: A Robust Preprocessing Pipeline for Functional MRI.” *Nature Methods* 16: 111–16. <https://doi.org/10.1038/s41592-018-0235-4>.

Evans, AC, AL Janke, DL Collins, and S Baillet. 2012. “Brain Templates and Atlases.” *NeuroImage* 62 (2): 911–22. <https://doi.org/10.1016/j.neuroimage.2012.01.024>.

Fonov, VS, AC Evans, RC McKinstry, CR Almli, and DL Collins. 2009. “Unbiased Nonlinear Average Age-Appropriate Brain Templates from Birth to Adulthood.” *NeuroImage* 47, Supplement 1: S102. <https://doi.org/10.1016/S1053-8119(09)70884-5>.

Gorgolewski, K., C. D. Burns, C. Madison, D. Clark, Y. O. Halchenko, M. L. Waskom, and S. Ghosh. 2011. “Nipype: A Flexible, Lightweight and Extensible Neuroimaging Data Processing Framework in Python.” *Frontiers in Neuroinformatics* 5: 13. <https://doi.org/10.3389/fninf.2011.00013>.

Gorgolewski, Krzysztof J., Oscar Esteban, Christopher J. Markiewicz, Erik Ziegler, David Gage Ellis, Michael Philipp Notter, Dorota Jarecka, et al. 2018. “Nipype.” *Software*. <https://doi.org/10.5281/zenodo.596855>.

Greve, Douglas N, and Bruce Fischl. 2009. “Accurate and Robust Brain Image Alignment Using Boundary-Based Registration.” *NeuroImage* 48 (1): 63–72. <https://doi.org/10.1016/j.neuroimage.2009.06.060>.

Jenkinson, Mark, Peter Bannister, Michael Brady, and Stephen Smith. 2002. “Improved Optimization for the Robust and Accurate Linear Registration and Motion Correction of Brain Images.” *NeuroImage* 17 (2): 825–41. <https://doi.org/10.1006/nimg.2002.1132>.

Klein, Arno, Satrajit S. Ghosh, Forrest S. Bao, Joachim Giard, Yrjö Häme, Eliezer Stavsky, Noah Lee, et al. 2017. “Mindboggling Morphometry of Human Brains.” *PLOS Computational Biology* 13 (2): e1005350. <https://doi.org/10.1371/journal.pcbi.1005350>.

Patriat, Rémi, Richard C. Reynolds, and Rasmus M. Birn. 2017. “An Improved Model of Motion-Related Signal Changes in fMRI.” *NeuroImage* 144, Part A (January): 74–82. <https://doi.org/10.1016/j.neuroimage.2016.08.051>.

Power, Jonathan D., Anish Mitra, Timothy O. Laumann, Abraham Z. Snyder, Bradley L. Schlaggar, and Steven E. Petersen. 2014. “Methods to Detect, Characterize, and Remove Motion Artifact in Resting State fMRI.” *NeuroImage* 84 (Supplement C): 320–41. <https://doi.org/10.1016/j.neuroimage.2013.08.048>.

Satterthwaite, Theodore D., Mark A. Elliott, Raphael T. Gerraty, Kosha Ruparel, James Loughead, Monica E. Calkins, Simon B. Eickhoff, et al. 2013. “An improved framework for confound regression and filtering for control of motion artifact in the preprocessing of resting-state functional connectivity data.” *NeuroImage* 64 (1): 240–56. <https://doi.org/10.1016/j.neuroimage.2012.08.052>.

Tustison, N. J., B. B. Avants, P. A. Cook, Y. Zheng, A. Egan, P. A. Yushkevich, and J. C. Gee. 2010. “N4ITK: Improved N3 Bias Correction.” *IEEE Transactions on Medical Imaging* 29 (6): 1310–20. <https://doi.org/10.1109/TMI.2010.2046908>.

Zhang, Y., M. Brady, and S. Smith. 2001. “Segmentation of Brain MR Images Through a Hidden Markov Random Field Model and the Expectation-Maximization Algorithm.” *IEEE Transactions on Medical Imaging* 20 (1): 45–57. <https://doi.org/10.1109/42.906424>.

**Supplementary Results**

To confirm that our primary results effects were robust to group differences between healthy controls and patients with peripheral nerve injury, we repeated our main analyses with healthy controls only (n=35, 66% of the total sample). This analysis found a similar overall pattern of results: inter-individual variability (parallel to **Figure 3**) showed a significant effect of Method (F_2,587_ = 18.19, p = 1.78 x 10^-7^), with significant pairwise differences of FSL-preproc < OGRE (p = 9.56 x 10^-10^), FSL-preproc < fMRIPrep (p = 0.001), and a trend toward OGRE > fMRIPrep (0.065). Z-scores in M1 parallel to **Figure 4B**) also showed a significant effect of Method (F(2,104) = 8.75, p = 3.1 x 10^-4^), with a significant pairwise effect of FSL-preproc < OGRE (p = 4.2 x 10^-4^), a trend toward FSL-preproc < fMRIprep (p = 0.057), and no difference between OGRE and fMRIPrep (p = 0.150). Therefore, despite the potential bias introduced by our unmodeled factor “patient group,” a subgroup analysis produces the same pattern of results: a consistent advantage for OGRE over FSL-preproc, and a less consistent advantage for OGRE over fMRIPrep.

**Supplementary Tables**

| **ROI #** | **FSL-preproc variability** | **OGRE variability** | **fMRIprep variability** |
| --- | --- | --- | --- |
| 1 | 1.408 | 1.103 | 1.446 |
| 2 | 1.056 | 1.140 | 0.931 |
| 3 | 1.074 | 1.436 | 0.865 |
| 4 | 2.052 | 2.308 | 1.051 |
| 5 | 0.787 | 1.094 | 0.541 |
| 6 | 0.829 | 3.025 | 0.834 |
| 7 | 0.378 | 1.002 | 0.463 |
| 8 | 0.397 | 0.429 | 0.345 |
| 9 | 0.557 | 0.498 | 0.397 |
| 10 | 0.864 | 1.279 | 0.701 |
| 11 | 0.478 | 0.556 | 0.507 |
| 12 | 0.807 | 0.854 | 0.707 |
| 13 | 0.674 | 0.441 | 0.542 |
| 14 | 0.596 | 0.510 | 0.740 |
| 15 | 0.661 | 0.574 | 0.619 |
| 16 | 0.611 | 0.523 | 0.646 |
| 17 | 0.713 | 0.659 | 0.780 |
| 18 | 1.533 | 1.327 | 1.418 |
| 19 | 1.923 | 1.978 | 1.779 |
| 20 | 0.543 | 0.437 | 0.564 |
| 21 | 0.611 | 0.515 | 0.587 |
| 22 | 0.398 | 0.458 | 0.473 |
| 23 | 1.056 | 0.867 | 0.611 |
| 24 | 0.352 | 0.312 | 0.402 |
| 25 | 0.614 | 0.477 | 0.530 |
| 26 | 0.327 | 0.316 | 0.343 |
| 27 | 0.687 | 0.474 | 0.558 |
| 28 | 0.609 | 0.592 | 0.699 |
| 29 | 0.515 | 0.532 | 0.646 |
| 30 | 0.414 | 0.366 | 0.449 |
| 31 | 1.337 | 1.127 | 1.407 |
| 32 | 0.605 | 0.483 | 0.570 |
| 33 | 0.738 | 0.679 | 0.497 |
| 34 | 0.392 | 0.391 | 0.464 |
| 35 | 0.666 | 0.524 | 0.446 |
| 36 | 0.776 | 0.631 | 0.629 |
| 37 | 0.419 | 0.347 | 0.442 |
| 38 | 0.510 | 0.475 | 0.646 |
| 39 | 0.468 | 0.425 | 0.515 |
| 40 | 0.735 | 0.728 | 0.825 |
| 41 | 0.500 | 0.444 | 0.538 |
| 42 | 0.478 | 0.381 | 0.460 |
| 43 | 0.390 | 0.406 | 0.371 |
| 44 | 0.482 | 0.458 | 0.487 |
| 45 | 0.494 | 0.355 | 0.554 |
| 46 | 0.598 | 0.549 | 0.603 |
| 47 | 0.311 | 0.283 | 0.337 |
| 48 | 0.518 | 0.467 | 0.576 |
| 49 | 0.512 | 0.508 | 0.535 |
| 50 | 0.494 | 0.461 | 0.515 |
| 51 | 0.404 | 0.302 | 0.414 |
| 52 | 0.265 | 0.195 | 0.246 |
| 53 | 0.892 | 0.909 | 0.569 |
| 54 | 0.283 | 0.293 | 0.368 |
| 55 | 0.560 | 0.407 | 0.579 |
| 56 | 0.837 | 0.610 | 0.919 |
| 57 | 0.600 | 0.426 | 0.672 |
| 58 | 0.379 | 0.385 | 0.404 |
| 59 | 0.295 | 0.243 | 0.299 |
| 60 | 0.270 | 0.202 | 0.286 |
| 61 | 0.451 | 0.336 | 0.481 |
| 62 | 0.331 | 0.273 | 0.334 |
| 63 | 0.587 | 0.470 | 0.616 |
| 64 | 0.453 | 0.447 | 0.463 |
| 65 | 0.615 | 0.517 | 0.591 |
| 66 | 0.363 | 0.303 | 0.420 |
| 67 | 0.590 | 0.537 | 0.505 |
| 68 | 0.387 | 0.260 | 0.250 |
| 69 | 0.460 | 0.407 | 0.415 |
| 70 | 0.293 | 0.258 | 0.308 |
| 71 | 0.484 | 0.463 | 0.573 |
| 72 | 0.758 | 0.672 | 0.840 |
| 73 | 0.465 | 0.374 | 0.427 |
| 74 | 0.399 | 0.406 | 0.457 |
| 75 | 0.307 | 0.235 | 0.321 |
| 76 | 0.431 | 0.424 | 0.555 |
| 77 | 0.269 | 0.237 | 0.255 |
| 78 | 0.378 | 0.254 | 0.320 |
| 79 | 0.507 | 0.373 | 0.512 |
| 80 | 0.444 | 0.438 | 0.545 |
| 81 | 0.485 | 0.588 | 0.414 |
| 82 | 0.719 | 0.718 | 0.651 |
| 83 | 0.406 | 0.336 | 0.420 |
| 84 | 0.278 | 0.240 | 0.297 |
| 85 | 0.319 | 0.316 | 0.383 |
| 86 | 0.359 | 0.252 | 0.341 |
| 87 | 0.492 | 0.370 | 0.551 |
| 88 | 0.513 | 0.527 | 0.450 |
| 89 | 0.410 | 0.326 | 0.414 |
| 90 | 0.386 | 0.363 | 0.429 |
| 91 | 0.558 | 0.479 | 0.458 |
| 92 | 0.723 | 0.640 | 0.639 |
| 93 | 0.591 | 0.462 | 0.440 |
| 94 | 0.483 | 0.378 | 0.412 |
| 95 | 0.848 | 0.588 | 0.736 |
| 96 | 1.351 | 1.013 | 0.763 |
| 97 | 0.323 | 0.226 | 0.260 |
| 98 | 0.472 | 0.378 | 0.440 |
| 99 | 0.332 | 0.234 | 0.279 |
| 100 | 0.476 | 0.455 | 0.360 |
| 101 | 0.814 | 0.620 | 0.623 |
| 102 | 0.322 | 0.220 | 0.309 |
| 103 | 0.339 | 0.281 | 0.330 |
| 104 | 0.445 | 0.378 | 0.483 |
| 105 | 0.546 | 0.393 | 0.504 |
| 106 | 0.901 | 0.880 | 1.066 |
| 107 | 0.510 | 0.394 | 0.580 |
| 108 | 0.608 | 0.425 | 0.514 |
| 109 | 0.506 | 0.380 | 0.489 |
| 110 | 0.522 | 0.436 | 0.569 |
| 111 | 1.234 | 0.718 | 0.933 |
| 112 | 0.467 | 0.362 | 0.470 |
| 113 | 0.463 | 0.347 | 0.399 |
| 114 | 0.923 | 0.596 | 0.863 |
| 115 | 0.439 | 0.326 | 0.473 |
| 116 | 0.515 | 0.354 | 0.405 |
| 117 | 0.525 | 0.558 | 0.461 |
| 118 | 0.412 | 0.293 | 0.385 |
| 119 | 0.377 | 0.271 | 0.369 |
| 120 | 0.836 | 0.728 | 0.538 |
| 121 | 0.492 | 0.430 | 0.367 |
| 122 | 0.310 | 0.268 | 0.290 |
| 123 | 0.502 | 0.377 | 0.478 |
| 124 | 0.367 | 0.308 | 0.290 |
| 125 | 0.425 | 0.333 | 0.338 |
| 126 | 0.568 | 0.440 | 0.481 |
| 127 | 0.353 | 0.318 | 0.341 |
| 128 | 0.338 | 0.309 | 0.361 |
| 129 | 0.854 | 0.671 | 0.588 |
| 130 | 0.461 | 0.340 | 0.452 |
| 131 | 0.269 | 0.221 | 0.280 |
| 132 | 0.360 | 0.413 | 0.382 |
| 133 | 0.314 | 0.374 | 0.242 |
| 134 | 0.523 | 0.536 | 0.521 |
| 135 | 0.253 | 0.231 | 0.241 |
| 136 | 0.659 | 0.559 | 0.612 |
| 137 | 0.551 | 0.411 | 0.510 |
| 138 | 0.510 | 0.460 | 0.541 |
| 139 | 0.582 | 0.516 | 0.626 |
| 140 | 0.512 | 0.380 | 0.502 |
| 141 | 1.095 | 0.856 | 1.077 |
| 142 | 1.146 | 0.861 | 1.181 |
| 143 | 1.829 | 1.801 | 1.727 |
| 144 | 1.256 | 1.406 | 1.081 |
| 145 | 0.659 | 0.750 | 0.741 |
| 146 | 0.857 | 0.825 | 0.781 |
| 147 | 0.754 | 0.699 | 0.673 |
| 148 | 0.561 | 0.449 | 0.636 |
| 149 | 0.736 | 0.673 | 0.732 |
| 150 | 0.677 | 0.589 | 0.727 |
| 151 | 0.547 | 0.557 | 0.559 |
| 152 | 0.488 | 0.385 | 0.403 |
| 153 | 0.736 | 0.715 | 0.579 |
| 154 | 0.623 | 0.503 | 0.492 |
| 155 | 1.194 | 0.994 | 0.734 |
| 156 | 0.796 | 0.682 | 0.823 |
| 157 | 0.730 | 0.670 | 0.789 |
| 158 | 0.841 | 0.716 | 0.825 |
| 159 | 0.911 | 0.743 | 0.897 |
| 160 | 1.010 | 0.892 | 0.896 |
| 161 | 0.998 | 0.781 | 0.680 |
| 162 | 0.550 | 0.419 | 0.506 |
| 163 | 0.432 | 0.312 | 0.392 |
| 164 | 0.477 | 0.389 | 0.448 |
| 165 | 0.471 | 0.444 | 0.409 |
| 166 | 0.775 | 0.673 | 0.796 |
| 167 | 1.041 | 1.084 | 0.990 |
| 168 | 0.528 | 0.431 | 0.544 |
| 169 | 0.526 | 0.426 | 0.598 |
| 170 | 0.630 | 0.491 | 0.718 |
| 171 | 0.609 | 0.521 | 0.598 |
| 172 | 0.455 | 0.349 | 0.457 |
| 173 | 0.528 | 0.425 | 0.465 |
| 174 | 1.111 | 1.338 | 1.050 |
| 175 | 0.548 | 0.522 | 0.441 |
| 176 | 0.544 | 0.408 | 0.535 |
| 177 | 0.552 | 0.429 | 0.522 |
| 178 | 0.471 | 0.395 | 0.402 |
| 179 | 0.390 | 0.323 | 0.322 |
| 180 | 0.335 | 0.265 | 0.313 |
| 181 | 0.654 | 0.538 | 0.798 |
| 182 | 0.408 | 0.362 | 0.327 |
| 183 | 0.638 | 0.482 | 0.505 |
| 184 | 0.610 | 0.591 | 0.618 |
| 185 | 0.884 | 0.595 | 0.709 |
| 186 | 0.480 | 0.442 | 0.562 |
| 187 | 0.824 | 0.926 | 0.854 |
| 188 | 0.529 | 0.374 | 0.430 |
| 189 | 0.588 | 0.495 | 0.515 |
| 190 | 0.974 | 0.994 | 0.609 |
| 191 | 0.576 | 0.448 | 0.523 |
| 192 | 0.447 | 0.366 | 0.441 |
| 193 | 0.355 | 0.319 | 0.354 |
| 194 | 0.543 | 0.481 | 0.524 |
| 195 | 0.605 | 0.530 | 0.554 |
| 196 | 0.904 | 0.862 | 0.984 |
| 197 | 0.400 | 0.322 | 0.357 |
| 198 | 0.490 | 0.419 | 0.535 |
| 199 | 1.017 | 1.008 | 0.798 |
| 200 | 0.351 | 0.299 | 0.325 |
| 201 | 0.427 | 0.404 | 0.437 |
| 202 | 0.414 | 0.310 | 0.403 |
| 203 | 0.447 | 0.370 | 0.418 |
| 204 | 0.304 | 0.215 | 0.297 |
| 205 | 0.679 | 0.548 | 0.767 |
| 206 | 0.699 | 0.524 | 0.669 |
| 207 | 0.629 | 0.461 | 0.667 |
| 208 | 0.328 | 0.252 | 0.319 |
| 209 | 0.508 | 0.464 | 0.435 |
| 210 | 0.272 | 0.341 | 0.320 |
| 211 | 0.313 | 0.228 | 0.358 |
| 212 | 0.545 | 0.407 | 0.498 |
| 213 | 0.335 | 0.284 | 0.294 |
| 214 | 0.321 | 0.242 | 0.343 |
| 215 | 0.547 | 0.347 | 0.489 |
| 216 | 0.446 | 0.370 | 0.383 |
| 217 | 0.577 | 0.478 | 0.469 |
| 218 | 0.534 | 0.461 | 0.445 |
| 219 | 0.419 | 0.324 | 0.389 |
| 220 | 0.627 | 0.562 | 0.692 |
| 221 | 0.693 | 0.880 | 0.594 |
| 222 | 0.544 | 0.410 | 0.522 |
| 223 | 0.418 | 0.373 | 0.438 |
| 224 | 0.638 | 0.521 | 0.696 |
| 225 | 0.505 | 0.491 | 0.477 |
| 226 | 0.649 | 0.554 | 0.617 |
| 227 | 0.628 | 0.450 | 0.556 |
| 228 | 1.344 | 0.917 | 0.719 |
| 229 | 1.439 | 0.887 | 0.806 |
| 230 | 0.783 | 0.753 | 0.796 |
| 231 | 0.980 | 0.805 | 0.829 |
| 232 | 0.639 | 0.625 | 0.764 |
| 233 | 0.628 | 0.675 | 0.744 |
| 234 | 0.940 | 0.945 | 0.985 |
| 235 | 1.124 | 1.081 | 1.293 |
| 236 | 0.484 | 0.404 | 0.523 |
| 237 | 0.399 | 0.381 | 0.524 |
| 238 | 0.231 | 0.189 | 0.252 |
| 239 | 0.250 | 0.194 | 0.250 |
| 240 | 0.509 | 0.463 | 0.531 |
| 241 | 1.118 | 1.169 | 0.822 |
| 242 | 0.899 | 0.899 | 0.848 |
| 243 | 0.391 | 0.503 | 0.488 |
| 244 | 0.570 | 0.694 | 0.653 |
| 245 | 0.546 | 0.626 | 0.600 |
| 246 | 0.515 | 0.476 | 0.379 |
| 247 | 0.478 | 0.514 | 0.502 |
| 248 | 0.451 | 0.309 | 0.385 |
| 249 | 0.370 | 0.263 | 0.363 |
| 250 | 0.494 | 0.370 | 0.514 |
| 251 | 0.431 | 0.301 | 0.407 |
| 252 | 0.372 | 0.357 | 0.359 |
| 253 | 0.340 | 0.393 | 0.347 |
| 254 | 0.332 | 0.428 | 0.379 |
| 255 | 0.421 | 0.376 | 0.435 |
| 256 | 0.409 | 0.368 | 0.377 |
| 257 | 0.397 | 0.402 | 0.409 |
| 258 | 0.363 | 0.302 | 0.339 |
| 259 | 0.315 | 0.275 | 0.300 |
| 260 | 0.311 | 0.293 | 0.313 |
| 261 | 0.354 | 0.316 | 0.341 |
| 262 | 0.857 | 0.789 | 0.978 |
| 263 | 0.763 | 0.707 | 0.803 |
| 264 | 0.317 | 0.265 | 0.305 |
| 265 | 0.301 | 0.245 | 0.279 |
| 266 | 0.515 | 0.431 | 0.493 |
| 267 | 0.430 | 0.371 | 0.438 |
| 268 | 0.337 | 0.247 | 0.298 |
| 269 | 0.371 | 0.255 | 0.315 |
| 270 | 0.334 | 0.262 | 0.303 |
| 271 | 0.358 | 0.328 | 0.370 |
| 272 | 0.370 | 0.358 | 0.392 |
| 273 | 0.327 | 0.265 | 0.310 |
| 274 | 0.529 | 0.654 | 0.668 |
| 275 | 0.520 | 0.462 | 0.494 |
| 276 | 0.440 | 0.383 | 0.509 |
| 277 | 0.636 | 0.495 | 0.631 |
| 278 | 0.619 | 0.550 | 0.615 |
| 279 | 0.732 | 0.453 | 0.507 |
| 280 | 0.389 | 0.276 | 0.341 |
| 281 | 0.350 | 0.348 | 0.398 |
| 282 | 0.324 | 0.279 | 0.358 |
| 283 | 0.607 | 0.444 | 0.651 |
| 284 | 0.462 | 0.338 | 0.562 |
| 285 | 0.437 | 0.320 | 0.420 |
| 286 | 0.623 | 0.529 | 0.495 |
| 287 | 0.559 | 0.497 | 0.557 |
| 288 | 0.510 | 0.404 | 0.434 |
| 289 | 0.384 | 0.284 | 0.373 |
| 290 | 0.412 | 0.345 | 0.456 |
| 291 | 0.488 | 0.378 | 0.448 |
| 292 | 0.524 | 0.442 | 0.502 |
| 293 | 0.746 | 0.956 | 1.089 |
| 294 | 0.451 | 0.517 | 0.558 |
| 295 | 0.584 | 0.520 | 0.545 |
| 296 | 0.604 | 0.560 | 0.643 |
| 297 | 0.419 | 0.276 | 0.367 |
| 298 | 0.444 | 0.378 | 0.459 |
| 299 | 0.324 | 0.221 | 0.292 |
| 300 | 0.401 | 0.289 | 0.403 |
| **Supplementary Table 1.** Variability (standard deviation across 53 participants) for each method and ROI. ROI definitions from Seitzman et al. 2020. | | | |

| **Mask Size GLMM 1**: outcome measure “BOLD standard deviation across ROIs” | | | | |
| --- | --- | --- | --- | --- |
| **Factor** | **Estimate** | **SE** | ***t*** | ***p*** |
| Intercept | -2.511 | 9.133 | -0.275 | 0.783 |
| Mask size | 1.41x10-5 | 4.04x10-5 | 0.348 | 0.728 |
| # non-brain voxels | -1.39x10-5 | 4.14x10-5 | -0.337 | 0.737 |
| Model | 0.039 | 0.236 | 0.166 | 0.868 |
| Mask Size * Model | -1.85x10-7 | 1.04x10-6 | -0.177 | 0.859 |
| NonBrain * Model | 2.28x10-7 | 1.07x10-6 | 0.213 | 0.831 |
| **Mask Size GLMM 2**: outcome measure “M1 Z-score” | | | | |
| **Factor** | **Estimate** | **SE** | ***t*** | ***p*** |
| Intercept | 126.39 | 142.68 | 0.886 | 0.377 |
| Mask size | -4.92x10-4 | 6.31x10-4 | -0.778 | 0.437 |
| # non-brain voxels | 4.87x1-04 | 6.47x11-04 | 0.753 | 0.452 |
| Model | -3.317 | 3.680 | -0.901 | 0.369 |
| Mask Size * Model | 1.48x10-5 | 1.53x10-5 | 0.909 | 0.365 |
| NonBrain * Model | -1.55x10-5 | 1.67x10-5 | -0.931 | 0.354 |
| **Supplementary Table 2.** GLMM results for effects of mask size (brain extraction results) on primary outcome measures. DF = 153 for all factors. | | | | |
